# Gender differences in the relationship between loneliness and health-related behavioral risk factors among the Hakka elderly in Fujian, China

**DOI:** 10.3389/fpsyt.2023.1196092

**Published:** 2023-06-02

**Authors:** Huajing Chang, Wenqian Ruan, Yating Chen, Longhua Cai, Xiaojun Liu

**Affiliations:** ^1^Department of Epidemiology and Health Statistics, School of Public Health, Fujian Medical University, Fuzhou, China; ^2^Department of Health Management, School of Health Management, Fujian Medical University, Fuzhou, China

**Keywords:** loneliness, health-related behavior, the Hakka elderly, gender differences, China

## Abstract

**Introduction:**

To explore gender differences in the relationship between loneliness and health-related behavioral risk factors (BRFs) among the Hakka elderly.

**Methods:**

Loneliness was measured by *the UCLA Loneliness Scale Short-form (ULS-8)*. Seven BRFs were examined. Mann–Whitney U, Kruskal-Wallis, and *post hoc* tests were conducted to compare the differences in ULS-8 scores among the Hakka elderly with different BRFs. Generalized linear regression models were employed to examine the associations of specific BRF and its number with the ULS-8 scores among the Hakka elderly in male, female, and total samples.

**Results:**

Physical inactivity (*B* = 1.96, *p* < 0.001), insufficient leisure activities participation (*B* = 1.44, *p* < 0.001), unhealthy dietary behavior (*B* = 1.02, *p* < 0.001), and irregular sleep (*B* = 2.45, *p* < 0.001) were positively correlated with the ULS-8 scores, whereas drinking (*B* = −0.71, *p* < 0.01) was negatively associated with the ULS-8 scores in the total sample. In males, insufficient leisure activities participation (*B* = 2.35, *p* < 0.001), unhealthy dietary behavior (*B* = 1.39, *p* < 0.001), and irregular sleep (*B* = 2.07, *p* < 0.001) were positively associated with the ULS-8 scores. In females, physical inactivity (*B* = 2.69, *p* < 0.001) and irregular sleep (*B* = 2.91, *p* < 0.001) was positively correlated with the scores of ULS-8, while drinking (*B* = −0.98, *p* < 0.05) was negatively associated with the ULS-8 scores. More BRFs were significantly related to greater loneliness (*p* < 0.001).

**Conclusion:**

There are gender differences in the relationship between loneliness and BRFs among the Hakka elderly, and individuals with more BRFs were more likely to feel loneliness. Therefore, the co-occurrence of multiple BRFs requires more attention, and integrated behavioral intervention strategies should be adopted to reduce the loneliness of the elderly.

## 1. Introduction

Population aging is a common challenge faced by most countries. According to the World Health Organization (WHO), the elderly population aged 60 and over is expected to reach 2 billion by 2050, accounting for 22% of the total population (1). Due to the decline in the birth rate and the increase in life expectancy, China became an aging society in 2000 (2). In 2021, China had 200 million people aged over 65 years (14.2% of the whole population) (3), and the number is forecast to double by 2050 (4). In contrast to developed countries such as Japan and the United States, China is experiencing “getting old before getting rich,” resulting in the emergence and accentuation of multiple health problems (5). Numerous studies observed gender differences in health status and most health problems (6). Women live longer but have higher rates of disability and poor health than men. Men have better health but die at a younger age than women. This phenomenon is known as the “male–female health-survival-paradox” (7). It is unclear whether Eastern populations, with different living conditions and cultures from contemporary Western societies, also experience gender differences in health. Therefore, exploring gender differences in health may have important implications for improving the health status and health outcomes of older adults in China.

Mental health is one of the most neglected global health issues, with approximately 15% of adults aged 60 and over suffering from a mental disorder (8). Loneliness is one of the key indicators of poor mental health, and people with existing mental health problems are also more likely to experience loneliness (9). In the context of rapid urbanization, changing family structures and the erosion of kinship ties in China, the prevalence of loneliness is gradually increasing, and 28% of older people have experienced loneliness (10). Gender differences in loneliness are also observed, but the results of existing studies are inconsistent. Some studies showed that men reported more intense feelings of loneliness than women, particularly those without a spouse or partner (11, 12). However, a WHO report noted that older women are vulnerable to loneliness, possibly related to longer lifespans and more severe health problems (8). In fact, for cultural reasons, older people, particularly men in China, may not receive timely treatment for loneliness because they are more reluctant to admit their loneliness. Therefore, investigating loneliness and its gender differences in older adults is of great significance for improving their overall health and quality of life.

Previous research demonstrated that there are associations between loneliness and health-related behavioral risk factors (BRFs) in older adults (13). Smoking, physical inactivity, and unhealthy diet were found to be significantly associated with a higher risk of loneliness (14). Evidence from longitudinal studies found that lonely individuals were less likely to engage in social interactions and physical activities (15), and more likely to smoke (10), drink alcohol (16), and experience sleep problems (17). What’s more, individuals with a co-occurrence of multiple health-related BRFs have been shown to be at higher risk of depression, anxiety, distress, and even chronic diseases and mortality than those with a single health-related risk behavior (18, 19). It’s also worth noting that the health-related BRFs were more likely to cluster in older men living in rural areas with lower socioeconomic status (20). However, there is a paucity of research investigating the association between the co-occurrence of BRFs and loneliness among the Chinese elderly, which means more attention is needed and essential.

The evidence for cultural differences in loneliness is complex, and people from different societies and cultures may experience varying degrees of loneliness (21). One study showed that living in a more collectivistic society was linked to greater loneliness (22). Han, the most populous ethnic group in China, has multiple internal ethnic branches, including the Hakka. There are around 80 million Hakkas worldwide, with 50 million residing in Guangdong, Jiangxi, and Fujian provinces in China. Compared with other Chinese, the Hakka people have distinct traditions and cultures. Previous studies showed that the Hakka people were forced to migrate from northern China to the south away from their homeland for historical reasons, which left them with limited access to resources (23). On the one hand, they gradually formed a living arrangement that a whole village lived together in a large “castled house” (tu lou), where people worked together and socialized closely. On the other hand, poor living conditions forced them to learn medical knowledge about various medicinal herbs and develop unique health-related behaviors to fight disease and maintain health in order to survive natural disasters such as extreme weather, poisons, and animal attacks. In recent years, with the change of lifestyle and the weakening of traditional filial piety, the traditional extended family has been gradually replaced by private nuclear families, and a rising number of the Hakka elderly live alone, which may put them at higher risk of loneliness and lead to some changes in their health-related BRFs. Therefore, this study aimed to explore the gender differences in the relationship between loneliness and health-related BRFs among the Hakka elderly. We hope to draw more attention to loneliness and health-related BRFs among Hakka older adults and to provide a theoretical basis for local governments to develop and implement relevant, effective, and targeted intervention programmes.

## 2. Materials and methods

### 2.1. Study design and participants

The present study is a cross-sectional, community-based survey conducted in Ninghua, Fujian, commonly known as the cradle of the Hakka. We collected data on the socio-ecological factors and mental health status of the Hakka elderly during the Spring Festival holidays in 2018. Theoretically, this study requires a minimum sample size of 385 using a 95% confidence level and a 5% margin of error. Our goal was to collect at least 1,000 valid samples. A convenience sampling strategy was adopted to increase enrolment during the specific data collection period. Our participants were residents aged 60 years or older, had a local household registration, and voluntarily participated in the survey. However, those who had a critical illness such as aphasia, deafness, blindness, and paraplegia, had severe mental disorders or dementia, or had a history of mental illness were excluded. Face-to-face interviews were conducted in consideration of the low education level among the elderly aged 60 or above in China. For those with limited ability to read and respond, we asked their family caregivers to help. Although we distributed a total of 1,500 paper-based questionnaires, some participants refused to participate at the very beginning, and some quit during the interview. Therefore, only 1,262 valid participants were included in the final analysis, with a valid survey response rate of 84.13%.

### 2.2. Measures

#### 2.2.1. General demographic characteristics

For this study, the general demographic characteristics included age, Hukou, current residence, education level, marital status, average annual household incomes (Chinese Yuan, CNY), living arrangement, and self-rated health status. Age was grouped into five categories, namely 60–64, 65–69, 70–74, 75–79, and ≥ 80 years. Participants were divided into three subgroups based on their marital status, namely married/cohabitation, widowed, and others (unmarried/divorced, separated, etc.). The setting of alternative options for participants’ sex, educational levels, and other variables is similar to *the China Health and Retirement Longitudinal Study (CHARLS)*, a nationally representative survey conducted by the National School of Development of Peking University (24). These general demographic characteristics were considered confounding factors for the association between health-related BRFs and loneliness among the Hakka elderly.

#### 2.2.2. Assessment of loneliness

We measured loneliness using *the University of California at Los Angeles (UCLA) Loneliness Scale Short-form (ULS-8)*, which is the most widely used instrument to assess loneliness (25). This scale consists of 6 items worded in a negative/lonely direction and 2 items worded in a positive/non-lonely direction. Each item is scored as 1 (never), 2 (rarely), 3 (sometimes), and 4 (always). The total ULS-8 scores range from 8 to 32 points, with higher scores indicating greater loneliness. The study used the Chinese version of the ULS-8, which was translated and validated by Zhou et al. (26). Before the formal survey, we conducted a pilot study to test the validity of the scale. In this study, Cronbach’s alpha coefficient for the ULS-8 was 0.949.

#### 2.2.3. Assessment of health-related risk behaviors

Seven health-related BRFs were measured in this study, including physical inactivity, insufficient leisure activities participation, unhealthy dietary behavior, current smoking, current drinking, irregular sleep, and unhealthy weight. According to the Healthy China Action Plan (2019–2030) (27) and the purpose of this study, health-related BRFs were defined as follows: (1) physical inactivity was defined if individuals did not meet the standard set by the Chinese Center for Disease Control and Prevention (CDC), i.e., exercising more than three times per week and for at least 30 min per time; (2) insufficient leisure activities participation was defined if individuals self-reported never participating in playing cards, mahjong, chess, etc.; (3) unhealthy dietary behavior was defined if individuals self-reported skipping breakfast or having an unbalanced diet such as insufficient intake of vegetables and fruit; (4) current smoking was defined if individuals self-reported smoking at least one cigarette per week, while current non-smokers were those who never smoked or had quit smoking; (5) current drinking was defined if individuals self-reported drinking more than once per week, and current non-drinkers were those who never drank or had quit drinking; (6) irregular sleep was defined if individuals self-reported sleeping less than 6 h or higher than 8 h per night (17); and (7) unhealthy weight was defined according to the body mass index (BMI) criteria for Chinese elderly from *the Chinese Nutrition Society (CNS)* (28), i.e., BMI < 18.5 kg/m^2^ or ≥ 24 kg/m^2^, and BMI was calculated from the height and weight of the participants obtained in the questionnaire. Finally, we calculated the total number of BRFs for each participant.

### 2.3. Statistical analysis

Statistical analysis was conducted using the Statistical Package for the Social Sciences (SPSS) version 25.0 for Windows (SPSS/IBM, Chicago, IL, United States). The alpha level was set at 5% as the criterion to determine statistical significance.

First, the demographic characteristics and BRFs variables were analyzed descriptively and reported as frequencies and proportions for the total, male and female samples. Next, the Mann–Whitney U’s test was used to compare the differences in ULS-8 scores between people with and without a BRF. The Kruskal-Wallis’s test was conducted to compare the differences in ULS-8 scores among three or more independent BRFs, and the *post hoc* tests were performed to explore specific differences in ULS-8 scores among the Hakka elderly with different numbers of BRF. Finally, generalized linear regression models were employed to examine the associations between the specific BRF and its number and the ULS-8 among the Hakka elderly in the male, female, and total samples. The final parsimonious model was adjusted for potential confounders. The unstandardized coefficients (B) with a 95% confidence interval (95% CI) obtained from the model were reported.

### 2.4. Ethical statement

Data collection for the present study was nested within a larger cross-sectional population-based survey known as *the China’s Health-Related Quality of Life Survey for Older Adults 2018 (CHRQLS-OA 2018)* (29). The study was conducted in accordance with the Declaration of Helsinki, and the study protocol was reviewed and approved by the Institutional Review Board of School of Health Science and Faculty of Medical Sciences, Wuhan University (IRB number: 2019YF2050). Informed consent information was included in each questionnaire and was introduced before the survey. Surveys were only conducted if participants were fully informed about the content and purpose of this research project and agreed to participate. The survey was also anonymous, and respondents’ information was kept confidential and used only for scientific research.

## 3. Results

### 3.1. Demographic characteristics of the study sample

A total of 1,262 Hakka older people were assessed in this study. As shown in Table 1, all subjects were divided into two groups. The number of males (48.57%) and females (51.43%) was comparable, with slightly more females included than males. Overall, the largest proportion of participants were aged between 60 and 64 years (28.21%), had an annual household income ranging from 15,001 to 30,000 CNY (27.65%), had an agricultural hukou (62.36%), were married or cohabited with others (66.80%), and self-rated health status as general good (56.34%). About 44.21% of male participants resided in counties, whereas 42.22% of female participants dwelt in villages. The majority of females (66.41%) were illiterate, while more than half of males (60.36%) had completed at least primary education. Males (38.66%) tended to live with their spouses only, while females (32.20%) were more likely to live with children.

**Table 1:** Demographic characteristics of the Hakka elderly (*n* = 1,262).

Variables	Total (*n* = 1,262)		Male (*n* = 613)		Female (*n* = 649)	
	*n*	%	*n*	%	*n*	%
Age (years)						
60–64	356	28.21	156	25.45	200	30.82
65–69	248	19.65	125	20.39	123	18.95
70–74	227	17.99	115	18.76	112	17.26
75–79	204	16.16	109	17.78	95	14.64
≥ 80	227	17.99	108	17.62	119	18.34
Hukou						
Agricultural Hukou	787	62.36	352	57.42	435	67.03
Non-agricultural Hukou	475	37.64	261	42.58	214	32.97
Current residence						
Village	478	37.88	204	33.28	274	42.22
Town	274	21.71	138	22.51	136	20.96
County	510	40.41	271	44.21	239	36.83
Education level						
Illiterate	674	53.41	243	39.64	431	66.41
Literacy class/home school	192	15.21	111	18.11	81	12.48
Primary school	192	15.21	107	17.46	85	13.10
≥ Junior high school	204	16.16	152	24.80	52	8.01
Marital status						
Married/cohabitation	843	66.80	442	72.10	401	61.79
Widowed	291	23.06	111	18.11	180	27.73
Others	128	10.14	60	9.79	68	10.48
Average household incomes (CNY)						
≤ 15,000	260	20.60	112	18.27	148	22.80
15,001 ~ 30,000	349	27.65	164	26.75	185	28.51
30,001 ~ 45,000	339	26.86	163	26.59	176	27.12
45,001 ~ 60,000	214	16.96	116	18.92	98	15.10
≥ 60,001	100	7.92	58	9.46	42	6.47
Living arrangement						
Living alone	104	8.24	34	5.55	70	10.79
Living with spouse only	400	31.70	237	38.66	163	25.12
Living with children	435	34.47	226	36.87	209	32.20
Mixed habitation	235	18.62	93	15.17	142	21.88
Others	88	6.97	23	3.75	65	10.02
Self-rated health status						
Very good/good	374	29.64	209	34.09	165	25.42
Fair	711	56.34	322	52.53	389	59.94
Very poor/poor	177	14.03	82	13.38	95	14.64

Categorical variables are presented as frequency (*n*) and percentage (%). CNY, Chinese Yuan.

### 3.2. Prevalence of BRFs in the study sample

Table 2 summarizes the prevalence of each BRF and the number of co-occurrent BRFs. Physical inactivity had the highest prevalence of 50.55%, while smoking had the lowest prevalence of 22.35%. Males were twice as likely to smoke and drink as females. Overall, the prevalence of multiple BRFs (i.e., 2 and more BRFs) was 66.63%. Notably, more than 90% of male participants had at least one or more BRFs.

**Table 2:** Prevalence of behavioral risk factors among the Hakka elderly.

Variables	Total		Male		Female	
	*n*	%	*n*	%	*n*	%
Behavioral risk factors						
Physical inactivity	638	50.55	289	47.15	349	53.78
Insufficient leisure activities participation	498	39.46	233	38.01	265	40.83
Unhealthy dietary behavior	586	46.43	271	44.21	315	48.54
Current smoking	282	22.35	206	33.61	76	11.71
Current drinking	553	43.82	352	57.42	201	30.97
Irregular sleep	563	44.61	240	39.15	323	49.77
Unhealthy weight	314	24.88	195	31.81	119	18.34
No. of behavioral risk factors						
0	160	12.68	44	7.18	116	17.87
1	261	20.68	130	21.21	131	20.18
2	218	17.27	136	22.19	82	12.63
3	189	14.98	89	14.52	100	15.41
4	165	13.07	59	9.62	106	16.33
5	122	9.67	63	10.28	59	9.09
6	129	10.22	78	12.72	51	7.86
7	18	1.43	14	2.28	4	0.62

Categorical variables are presented as frequency (n) and percentage (%).

### 3.3. ULS-8 scores of study sample

We compared the ULS-8 scores of participants with different BRFs (Table 3). The Mann–Whitney *U*’s test indicated that elderly participants with the behavior of physical inactivity (*Z* = −23.363, *p* < 0.001), insufficient leisure activities participation (*Z* = −21.041, *p* < 0.001), unhealthy dietary behavior (*Z* = −14.020, *p* < 0.001), smoking (*Z* = −8.630, *p* < 0.001) and irregular sleep (*Z* = −21.436, *p* < 0.001) had significantly higher (i.e., worse) ULS-8 scores than those without these behaviors. In the total sample, there was no significant difference in ULS-8 scores between the Hakka elderly with unhealthy weight and healthy weight. However, male participants with unhealthy weight were found to have significantly lower ULS-8 scores than those with healthy weight (*p* < 0.001), while female participants with unhealthy weight had higher ULS-8 scores (*p* < 0.001).

**Table 3:** Comparison of ULS-8 scores for BRFs among the Hakka elderly.

Variables		Total	Male	Female
		Mean ± S. D	Mean ± S. D	Mean ± S. D
Behavioral risk factors				
Physical inactivity	Yes	20.73 ± 6.06	19.58 ± 6.12	21.68 ± 5.85
	No	11.71 ± 3.85	11.35 ± 3.77	12.11 ± 3.90
	*Z*	−23.363^***^	−15.789^***^	−17.107^***^
Insufficient leisure activities participation	Yes	21.25 ± 5.68	20.50 ± 5.79	21.90 ± 5.50
	No	13.03 ± 5.36	12.00 ± 4.45	14.05 ± 5.96
	*Z*	−21.041^***^	−15.654^***^	−14.089^***^
Unhealthy dietary behavior	Yes	19.24 ± 6.84	18.07 ± 6.63	20.24 ± 6.88
	No	13.70 ± 5.61	12.98 ± 5.40	14.45 ± 5.73
	*Z*	−14.020^***^	−9.155^***^	−10.441^***^
Current smoking	Yes	19.50 ± 6.37	18.98 ± 6.52	20.91 ± 5.74
	No	15.34 ± 6.64	13.33 ± 5.58	16.77 ± 6.95
	*Z*	−8.630^***^	−9.733***	−4.572^***^
Current drinking	Yes	16.09 ± 6.83	15.67 ± 6.80	16.83 ± 6.83
	No	16.41 ± 6.78	14.64 ± 5.99	17.45 ± 7.00
	*Z*	−1.820	−1.045	−1.405
Irregular sleep	Yes	20.98 ± 6.20	19.90 ± 6.20	21.78 ± 6.08
	No	12.48 ± 4.51	12.23 ± 4.63	12.78 ± 4.36
	*Z*	−21.546^***^	−13.824^***^	−16.177^***^
Unhealthy weight	Yes	16.10 ± 6.83	13.55 ± 5.33	20.27 ± 6.97
	No	16.33 ± 6.79	16.01 ± 6.82	16.58 ± 6.76
	*Z*	−0.791	−3.494^***^	−4.981^***^
No. of behavioral risk factors				
0 (g1)		11.18 ± 2.06	10.16 ± 1.03	11.57 ± 2.21
1 (g2)		11.37 ± 3.86	10.58 ± 2.75	12.15 ± 4.59
2 (g3)		12.50 ± 4.48	11.73 ± 3.95	13.79 ± 4.99
3 (g4)		19.39 ± 6.35	17.17 ± 6.18	21.37 ± 5.85
4 (g5)		21.76 ± 5.46	20.05 ± 5.53	22.71 ± 5.21
5 (g6)		21.54 ± 5.85	19.90 ± 6.16	23.29 ± 4.98
6 (g7)		21.45 ± 6.05	21.17 ± 6.13	21.88 ± 5.96
7 (g8)		22.44 ± 3.78	21.64 ± 3.93	25.25 ± 0.50
*H*		586.143^***^	278.659^***^	331.612^***^
Pairwise Comparison		g8 & g7 & g6 & g5 & g4 > g3 & g2 & g1	g8 & g7 & g6 & g5 & g4 > g3 & g2 & g1, g7 > g4	g8 & g7 & g5 & g4 > g2 & g1, g7 & g6 & g5 & g4 > g3

^*^ *p*-value < 0.05, ^**^ *p*-value < 0.01 and ^***^ *p*-value < 0.001. S. D, standard deviation.

Kruskal-Wallis’s analysis revealed that the mean values of ULS-8 scores were significantly different among individuals with different numbers of BRFs (*p* < 0.001). The ULS-8 scores increased with the increase of the number of BRFs. The results of pairwise comparison illustrated that the elderly participants with 8 BRFs obtained the highest ULS-8 scores.

### 3.4. Association between BRFs and ULS-8 scores

The final parsimonious model (model 2) demonstrated that, in the total sample, physical inactivity (*B* = 1.96, 95% *CI* = 1.19 to 2.73, *p* < 0.001), insufficient leisure activities participation (*B* = 1.44, 95% *CI* = 0.75 to 2.13, *p* < 0.001), unhealthy dietary behavior (*B* = 1.02; 95% *CI* = 0.55 to 1.50, *p* < 0.001), and irregular sleep (*B* = 2.45, 95% *CI* = 1.88 to 3.03, *p* < 0.001) were positively correlated with the ULS-8 scores, whereas drinking (*B* = −0.71, 95% *CI* = −1.20 to −0.22, *p* < 0.01) was negatively associated with the ULS-8 scores. In the male sample, insufficient leisure activities participation (*B* = 2.35, 95% *CI* = 1.38 to 3.32, *p* < 0.001), unhealthy dietary behavior (*B* = 1.39, 95% *CI* = 0.76 to 2.02, *p* < 0.001) and irregular sleep (*B* = 2.07, 95% *CI* = 1.29 to 2.84, *p* < 0.001) were positively associated with the ULS-8 scores. In the female sample, physical inactivity (*B* = 2.69, 95% *CI* = 1.49 to 3.89, *p* < 0.001) and irregular sleep (*B* = 2.91, 95% *CI* = 2.02 to 3.79, *p* < 0.001) were positively correlated with the scores of ULS-8, while drinking (*B* = −0.98, 95% *CI* = −1.72 to −0.23, *p* < 0.05) was negatively associated with the ULS-8 scores (Table 4).

**Table 4:** Generalized linear regression models testing the association between BRFs and ULS-8 scores.

Behavioral risk factors	Total		Male		Female	
	*B*	95% *CI*	*B*	95% *CI*	*B*	95% *CI*
Model 1						
Physical inactivity	4.35	(3.51, 5.20)^***^	2.83	(1.66, 3.99)^***^	5.64	(4.44, 6.84)^***^
Insufficient leisure activities participation	2.38	(1.58, 3.18)^***^	3.72	(2.56, 4.88)^***^	1.01	(−0.07, 2.09)
Unhealthy dietary behavior	1.69	(1.13, 2.25)^***^	1.71	(0.94, 2.48)^***^	1.38	(0.58, 2.18)^**^
Current smoking	−0.45	(−1.15, 0.26)	0.70	(−0.23, 1.63)	−1.39	(−2.60, −0.18)^*^
Current drinking	−0.72	(−1.27, −0.17)^*^	−0.42	(−1.18, 0.34)	−0.71	(−1.50, 0.08)
Irregular sleep	4.32	(3.67, 4.97)^***^	3.30	(2.37, 4.22)^***^	4.74	(3.82, 5.66)^***^
Unhealthy weight	0.03	(−0.55, 0.61)	−0.43	(−1.20, 0.34)	1.10	(0.18, 2.01)^*^
Model 2						
Physical inactivity	1.96	(1.19, 2.73)^***^	0.74	(−0.29, 1.76)	2.69	(1.49, 3.89)^***^
Insufficient leisure activities participation	1.44	(0.75, 2.13)^***^	2.35	(1.38, 3.32)^***^	0.60	(−0.39, 1.59)
Unhealthy dietary behavior	1.02	(0.55, 1.50)^***^	1.39	(0.76, 2.02)^***^	0.61	(−0.12, 1.33)
Current smoking	−0.04	(−0.66, 0.58)	−0.42	(−1.19, 0.35)	0.52	(−0.61, 1.64)
Current drinking	−0.71	(−1.20, −0.22)^**^	−0.38	(−1.01, 0.24)	−0.98	(−1.72, −0.23)^*^
Irregular sleep	2.45	(1.88, 3.03)^***^	2.07	(1.29, 2.84)^***^	2.91	(2.02, 3.79)^***^
Unhealthy weight	0.16	(−0.35, 0.67)	−0.05	(−0.70, 0.60)	0.38	(−0.45, 1.22)

B = Unstandardized Coefficient, ^*^ *p*-value < 0.05, ^**^ *p*-value < 0.01 and ^***^ *p*-value < 0.001. Model 1 is adjusted for all behavioral risk factors. Model 2 is adjusted for sociodemographic variables, like age, Hukou, current residence, etc.

### 3.5. Association between the number of BRFs and ULS-8 scores

We further examined whether the number of BRFs was related to loneliness among the Hakka elderly (Table 5). Overall, after controlling for other covariates (sociodemographic characteristics), more BRFs were significantly related to greater loneliness. In particular, as compared with the reference group (people with zero BRF), individuals with three BRFs (*B* = 3.28, 95% *CI* = 2.34 to 4.22, *p* < 0.001), four BRFs (*B* = 4.55, 95% *CI* = 3.56 to 5.55, *p* < 0.001), five BRFs (*B* = 4.00, 95% *CI* = 2.93 to 5.07, *p* < 0.001), six BRFs (*B* = 4.59, 95% *CI* = 3.52 to 5.65, *p* < 0.001) and seven BRFs (*B* = 4.92, 95% *CI* = 2.92 to 6.93, *p* < 0.001) showed a higher ULS-8 scores.

**Table 5:** Generalized linear regression models testing the association between the number of BRFs and ULS-8 scores.

No. of behavioral risk factors	Total		Male		Female	
	*B*	95% *CI*	*B*	95% *CI*	*B*	95% *CI*
Model 1						
0	–	–	–	–	–	–
1	0.19	(−0.78, 1.15)	0.42	(−1.20, 2.03)	0.58	(−0.61, 1.78)
2	1.32	(0.32, 2.33)^*^	1.57	(−0.04, 3.18)	2.22	(0.87, 3.57)^**^
3	8.21	(7.18, 9.24)^***^	7.01	(5.30, 8.72)^***^	9.80	(8.53, 11.08)^***^
4	10.58	(9.51, 11.64)^***^	9.89	(8.05, 11.74)^***^	11.14	(9.88, 12.39)^***^
5	10.36	(9.20, 11.52)^***^	9.75	(7.92, 11.57)^***^	11.72	(10.22, 13.21)^***^
6	10.27	(9.13, 11.41)^***^	11.01	(9.26, 12.76)^***^	10.31	(8.74, 11.88)^***^
7	11.26	(8.87, 13.66)^***^	11.48	(8.64, 14.33)^***^	13.68	(8.93, 18.44)^***^
Model 2						
0	–	–	–	–	–	–
1	−0.56	(−1.34, 0.22)	0.48	(−0.82, 1.78)	−0.51	(−1.56, 0.54)
2	−0.19	(−1.03, 0.66)	0.16	(−1.13, 1.46)	−0.18	(−1.38, 1.02)
3	3.28	(2.34, 4.22)^***^	2.61	(1.18, 4.05)^***^	4.65	(3.31, 5.98)^***^
4	4.55	(3.56, 5.55)^***^	4.10	(2.51, 5.69)^***^	5.08	(3.74, 6.42)^***^
5	4.00	(2.93, 5.07)^***^	3.22	(1.62, 4.82)^***^	5.29	(3.79, 6.79)^***^
6	4.59	(3.52, 5.65)^***^	4.91	(3.34, 6.49)^***^	4.57	(3.00, 6.14)^***^
7	4.92	(2.92, 6.93)^***^	4.12	(1.73, 6.51)^**^	6.66	(2.66, 10.66)^**^

B = Unstandardized Coefficient, ^*^ *p*-value < 0.05, ^**^ *p*-value < 0.01 and ^***^ *p*-value < 0.001. Model 1 is the crude model. Model 2 is adjusted for sociodemographic variables like age, Hukou, current residence, etc.

## 4. Discussion

The word “Hakka” means “guests,” “foreigners,” or “strangers,” which is related to the history of their multiple migrations, implying that they are not the original inhabitants of the region (23). Therefore, although the Hakka is an ethnic branch of Han Chinese, their lifestyles, beliefs, and culture are in part distinct from those of ordinary Chinese people. This study found that the Hakka elderly who had physical inactivity, insufficient leisure activities participation, unhealthy dietary behavior, smoking, or irregular sleep behaviors had greater loneliness, which is consistent with other studies (30, 31). Surprisingly, although the unhealthy weight was associated with loneliness in both male and female Hakka elderly groups, we found that males with unhealthy weight had significantly lower ULS-8 scores than those with healthy weight, while females with unhealthy weight had significantly higher ULS-8 scores, which caused the relationship between unhealthy weight and loneliness in the total population not statistically significant. Possible explanations for this phenomenon are as follows: Firstly, females are more likely to maintain a healthy weight because of the body size stereotype, and obese people are more likely to face stigma and discrimination. Secondly, due to the traditional Chinese idea that “body fat represents rich” and better living conditions nowadays, some older adults, especially those who survived the “Great Famine” in the 1960s, may mitigate the effects of loneliness by consuming more food (32), and this phenomenon is more evident in males (33). Thirdly, an unhealthy weight may be a sign of poor health status. Previous studies showed that individuals with underweight (34), overweight, or obese (33) are more likely to have mental health issues than those with a healthy weight. Compared to the male elderly, the female elderly had higher rates of disability and poor health (7), which could further cause unhealthy weight and loneliness issues.

Physical activity can help to combat loneliness by improving people’s moods and developing their social networks and social capital (35). Studies showed that loneliness was pervasive among physically inactive persons (36), particularly in middle-aged populations (37). Varied effects of physical activities were also found on males and females (38). Our result showed that a positive association occurred only in the Hakka female elderly, which may be because females are better at building friendships in the process of participating in exercise (38). But in fact, more than half of the Hakka female elderly who participated in our survey lacked physical activity. This may be explained by the low socioeconomic status of Chinese women and the poorer motor function of females than males (7). Compared with ordinary Chinese elderly, Hakka elderly people have a unique living arrangement. They usually live in villages or family units in “tulou” and have closer social networks and bonding social capital, which means that mobilizing Hakka older people to participate in physical activity may have a significant effect on the improvement of loneliness. In the past few years, square dancing has been a particularly popular activity among the Chinese elderly, especially females (39). Therefore, government agencies could encourage the Hakka female elderly to engage in physical activities by expanding sports venues and increasing sports equipment for physical activities like square dancing to reduce their loneliness.

Our findings showed a negative correlation between leisure activity and loneliness among the total Hakka older population. The effectiveness of leisure activities in preventing and alleviating loneliness among the elderly has been proven (40). Participation in leisure activities is important for social interaction and social contact among the elderly in China. For older persons who have retired, leisure activities can provide them some opportunities to meet people and engage in social events, which could meet their psychological needs (41). Our study also found that the negative association between leisure activities and loneliness was significant for older males but not for older females. Insufficient participation in leisure activities could leave males with fewer social interaction opportunities and narrower social networks (42), which may cause negative emotions such as loneliness, depression, etc. In China, the participation rate in leisure activity among the elderly has shown a downward trend over the past two decades (43). The Hakka people have traditionally been dominated by a culture of extended family. Older males, who were often the most authoritative members of the extended family, may suffer more loneliness from the changes of children leaving home and declining social status. Therefore, we should advocate strengthening the monitoring of engagement in leisure activity among the Hakkas, particularly the Hakka male elderly, and call for more research on the negative trends of leisure activities.

Unhealthy diets are currently becoming a major threat to the health of Chinese people. Previous studies showed that the prevalence of unhealthy diet was highest among people aged 65 years and older (44). Our results showed that unhealthy dietary behavior was strongly correlated with loneliness in the total and male population, which was consistent with previous studies (45). With the population migrating and life expectancy increasing, rural hollowing out is becoming more serious, and the number of empty-nest elderly is gradually increasing. One study showed that the empty-nest elderly were more prone to loneliness and depression than the nonempty-nest elderly (28). Older men living alone had significantly worse eating patterns and more unhealthy eating behaviors than those living with a spouse (29). On the one hand, the Hakka elderly may eat their favorite but unhealthy foods to alleviate loneliness. On the other hand, unhealthy diets may lead to a variety of physical and psychological problems and increase the prevalence of chronic diseases, which indirectly leads to a stronger sense of loneliness among the Hakka elderly. Unfortunately, this study did not explore the differences between the specific dietary styles of the Hakka elderly and the general Chinese elderly. Therefore, we should further study the association between the dietary situation and the mental health status of the Hakka elderly, and government departments should focus on the empty-nest elderly living alone when formulating and implementing policies.

It is difficult to come to a firm conclusion about the relationship between drinking and loneliness. Some studies suggested that lonely persons tend to drink excessively (46, 47), while other studies found that loneliness is associated with lower alcohol consumption (48, 49). Our findings observed a negative association between drinking and loneliness in the Hakka elderly. There are some possible explanations: On the one hand, drinking is considered an essential social activity in Chinese culture. A study of retired older adults found that adults who were socially isolated in their retirement communities were less likely to drink regularly and heavily than those who were more socially connected (50). A stronger sense of belonging brought by group drinking was found to be associated with reduced loneliness (48). The Hakka elderly have fewer opportunities to socialize, which decreases their access to alcohol intake but heightens their sense of loneliness. On the other hand, the unique socio-ecological environment of the Hakka area has shaped a winemaking culture, and the Hakkas’ tolerance, openness, and hospitality are reflected in their drinking culture, allowing them to drink more alcohol but helping to feel less lonely. Thus, it is hard to distinguish whether loneliness prompted the Hakkas who initially drank to drink less or whether the drinking habit reduced loneliness in the current cross-sectional study. Moreover, there is considerable evidence that alcohol consumption is detrimental to health (51), and it would be unwise to conclude that high alcohol intake can reduce loneliness based on our findings alone. Therefore, further research is warranted to understand the underlying physiological and psychosocial connection between loneliness and drinking, which may assist in the development of more effective interventions and preventative measures for loneliness.

In China, the prevalence of sleep problems has been reported to range from 8.3 to 49.7% (52). Older people who felt lonely were found to have a higher prevalence of sleep-related issues, such as insomnia and sleep deprivation (17, 31). This may be because sleep is particularly sensitive to psychological stressors (53). Our study found that irregular sleep was positively associated with loneliness in the total, male and female Hakka elderly, and this is in line with previous studies (17, 31, 53, 54). As a matter of fact, the brain of lonely people maintains a certain level of alertness during sleep, which leads to a decrease in sleep quality and an increase in depression (54). What’s worse, sleep problems could further increase people’s loneliness. Patients with loneliness display a foul mood or low mood throughout the day due to poor sleep quality, numerous nighttime awakenings, daytime sleepiness, and exhaustion (55). In summary, all the evidence suggests that improving sleep health is crucial to the prevention and treatment of loneliness, and further empirical studies are needed. Hakka people have unique beliefs and culture. They believe in gods and have special reverence and worship for the sky, land, flora and fauna. Worshiping the gods can help them relieve anxiety, sadness, pain and bring peace of mind. It can be considered from this point to improve the sleep problems and loneliness of the Hakka elderly.

We also found that the number of BRFs was strongly positively associated with loneliness among the Hakka elderly after adjusting for confounding factors. Besides, there are some differences in the number of BRFs among the Hakka elderly by gender, and most of them had at least one health-related risk behavior. One study showed that about 57.0% of Chinese people had at least two BRFs, and the prevalence was higher among older men, those living in rural areas, and those with lower socioeconomic status (24). Another evidence suggested that those with lower socioeconomic or educational levels were four to five times more likely to have multiple BRFs (56). However, existing behavioral interventions remain focused on preventing and controlling of single health-related risk behavior, ignoring the potential linkages between multiple BRFs. Previous studies indicated implementing interventions that address multiple risk factors simultaneously is more effective than interventions that target a single risk factor (57). Therefore, comprehensive interventions to address multiple BRFs should be quickly planned and implemented to reduce health-related risk behaviors and improve the loneliness of the Hakka elderly.

## 5. Conclusion

In conclusion, the present study has gained an overall understanding of the relationship between health-related risk behaviors and loneliness among the Hakka elderly in Fujian. Our study showed that the Hakka elderly with BRFs were more likely to feel lonely than those without BRFs, except for drinking and unhealthy weight. After adjusting for social-demographic variables, we found that factors such as insufficient leisure activities participation, unhealthy dietary behavior, and irregular sleep were positively associated with loneliness among the Hakka male elderly. In the Hakka female elderly, physical inactivity and irregular sleep were positively associated with loneliness, while drinking was negatively related to loneliness. Furthermore, the association between the number of BRFs and loneliness was strongly positively correlated, and individuals with more BRFs were more likely to self-report loneliness. Hence, we recommend that when formulating health policies and intervention measures, government departments should not only pay attention to specific single health-related BRFs according to gender, but also explore the co-occurrence of multiple BRFs, and adopt comprehensive strategies to reduce the loneliness of the elderly.

## 6. Limitation

To the best of our knowledge, this study is the first research targeting the Hakka elderly to reveal the relationship between their BRFs and loneliness. The following limitations of the present study should be noted: Firstly, only correlation rather than causal relationship was explored due to the cross-sectional design of our study, so further prospective research is needed. Secondly, our study only investigated the Hakka elderly in Fujian, China. However, the Hakka people are also distributed in Guangdong, Jiangxi in China, and other Southeast Asian countries like Thailand, Malaysia, and Singapore. Our samples cannot well summarize the entire Hakka elderly population. Therefore, the conclusions of this study need to be extrapolated to other populations with caution, and it is necessary to conduct further comparative studies between the Hakka elderly and the ordinary Chinese elderly in the future. Thirdly, non-response bias was not assessed, as only those who agreed to participate were included in the analyses. Lastly, there are differences in the patterns of behavior co-occurrence, and this study did not explore the impact of such differences on loneliness. It is necessary for future studies to use methods such as clustering or latent class analysis to identify behavior patterns and explore their relationship with loneliness.

## Data availability statement

The raw data supporting the conclusions of this article will be made available by the authors, without undue reservation.

## Author contributions

XL conceived this research. HC and WR were responsible for the methodology. HC and WR conducted software analyses, gathered resources, curated all data, wrote and prepared the original draft. XL was responsible for project administration. YC and LC conducted necessary validations. XL conducted a formal analysis and managed the investigation. XL reviewed and edited the manuscript, was responsible for visualization, supervised the project, and acquired the funding. All authors contributed to the article and approved the submitted version.

## Funding

The present study was mainly supported by the National Natural Science Foundation of China (Grant No. 72204047). Meanwhile, this work was also partially supported by the Natural Science Foundation of Fujian Province, China (Grant No. 2022 J01234).

## Conflict of interest

The authors declare that the research was conducted in the absence of any commercial or financial relationships that could be construed as a potential conflict of interest.

## Publisher’s note

All claims expressed in this article are solely those of the authors and do not necessarily represent those of their affiliated organizations, or those of the publisher, the editors and the reviewers. Any product that may be evaluated in this article, or claim that may be made by its manufacturer, is not guaranteed or endorsed by the publisher.
